# From Cellular Attractor Selection to Adaptive Signal Control for Traffic Networks

**DOI:** 10.1038/srep23048

**Published:** 2016-03-14

**Authors:** Daxin Tian, Jianshan Zhou, Zhengguo Sheng, Yunpeng Wang, Jianming Ma

**Affiliations:** 1 Beihang University, School of Transportation Science and Engineering, Beijing Key Laboratory for Cooperative Vehicle Infrastructure Systems and Safety Control, Beijing, 100191, China; 2 University of Sussex, Department of Engineering and Design, Brighton, BN1 9RH, United Kingdom; 3 The Texas Department of Transportation, 10016 Liriope Cove, Austin, TX 78750, United States; 4 Jiangsu Province Collaborative Innovation Center of Modern Urban Traffic Technologies, No. 2 SiPaiLou, Nanjing, 210096, China

## Abstract

The management of varying traffic flows essentially depends on signal controls at intersections. However, design an optimal control that considers the dynamic nature of a traffic network and coordinates all intersections simultaneously in a centralized manner is computationally challenging. Inspired by the stable gene expressions of *Escherichia coli* in response to environmental changes, we explore the robustness and adaptability performance of signalized intersections by incorporating a biological mechanism in their control policies, specifically, the evolution of each intersection is induced by the dynamics governing an adaptive attractor selection in cells. We employ a mathematical model to capture such biological attractor selection and derive a generic, adaptive and distributed control algorithm which is capable of dynamically adapting signal operations for the entire dynamical traffic network. We show that the proposed scheme based on attractor selection can not only promote the balance of traffic loads on each link of the network but also allows the global network to accommodate dynamical traffic demands. Our work demonstrates the potential of bio-inspired intelligence emerging from cells and provides a deep understanding of adaptive attractor selection-based control formation that is useful to support the designs of adaptive optimization and control in other domains.

Signalised intersections, which can be treated as a set of networked control junctions that play a significant role in managing varying flows in a traffic network, are ubiquitous in urban areas^1^. In a signal timing plan, the design of a suitable stage duration and sequence is a key issue. Due to the computational costs arising from either the increasing number of links and junctions involved in a traffic network or additional traffic-predicting information requested in embedded traffic models, many adaptive controls based on some existing computational intelligence methods such as ADP (Approximated Dynamical Programming), enhanced learning (Q-learning), Markov chain-based decision, artificial neural networks, fuzzy logic algorithms and control theories simply focus on an isolated intersection or the simplified local structure of a network^2^,^3^,^4^,^5^,^6^,^7^,^8^,^9^. For traffic networks of multiple intersections, many researchers have been engaged in developing adaptive and self-organized paradigms within various domains, such as the statistical mechanics, the physics and the operations research, etc. Among these, the cellular automata based approaches have attracted much attention^10^,^11^,^12^,^13^. Besides, a decentralized traffic light control method inspired by the self-organization in pedestrian counter-flows at bottlenecks was proposed in^14^, and a dynamic programming principle method was proposed in^15^. Some self-organizing traffic light controls were developed based on the synchronization strategies of coupled oscillators^16^,^17^,^18^. These synchronization strategies model each signalized intersection as an oscillator and perform local interactions between oscillators in order to achieve collective behavior. Generally, there are three major challenges to realise a real-time self-adaptive distributed signal control: (i) the highly dynamic, stochastic and nonlinear nature of traffic demands or traffic loads on roads; (ii) the increased computational cost and complexity of a large-scale traffic network and (iii) the impracticality or even absence of centralised infrastructure for coordinating global signalised intersections in real life. Clearly, these challenges are common and intrinsic in traffic networks as a typical class of artificial control systems, which have already gone far beyond what conventional optimisation and control paradigms can do for the deployment, management and maintenance of complex signalised intersections. In fact, most existing technological architecture cannot accommodate multiple aspects along with the evolution of traffic signal systems including randomness, complexity, scalability and other factors simultaneously. At this point, we pose an essential question: are there any mechanisms or design principles that can induce global signalised intersections to dynamically self-adapt to the varying traffic conditions of their network in a fully-distributed and autonomous manner? To answer this question, we refer to nature and look to biology as a key source of inspiration. Here we exploit a certain biological adaptive mechanism on a micro level (i.e. on a cellular basis) and also employ a relevant mathematical model that presents the dynamics of cells’ stable gene expressions in adaptation to varying environmental conditions. Our study demonstrates how such a biological characteristic inherent in cells’ genetic programs can be applied to design an innovative, simple and adaptive signal-control paradigm to address, to a certain extent, those aforementioned challenges in order to realise intelligent traffic networks; more importantly, it helps to deepen the understanding of cell-inspired intelligence that may be a promising inspiration for other optimisation and control applications.

As the basic functional unit of life, cells are biologically simple structures. Even so, as an outcome of billions of years of natural evolution, cells have come to possess some appealing biological characteristics, enabling them to be resilient to external damage and robust against biological noises, to adapt to varying environmental conditions and to infer their environmental state to make smart decisions^19^,^20^,^21^,^22^,^23^. In addition, fully-distributed autonomy and self-organisation can also emerge from the simple-rules-based interactions of populations of cells, which allows them to utilise constrained environmental resources with high efficiency, in order to co-exist and co-evolve^24^,^25^. These facts can lead to various inspirations on the microbiological level towards novel designs for effective solutions to certain engineering problems, as shown by some successful research cases^26^,^27^,^28^,^29^. In light of these successes, our investigation also follows a biological inspiration-based approach to propose a novel dynamical and adaptive signal-control method for traffic networks. Specifically, we employ a biological mechanism intrinsic to the adaptive behaviour of the *Escherichia coli* (*E. coli*) cells known as attractor selection, which can induce the cellular gene network to dynamically accommodate its genetic programs to changes in environmental conditions^30^. Accordingly, an adaptive attractor corresponds to a type of stable genetic program. Thus, *E. coli* cells usually prefer to switch to a stable genetic program, i.e. selecting an adaptive attractor, so as to survive better (yielding a better metabolic phenotype) in a new external environment after the environmental conditions (such as nutrients) have been changed. Recently, this attractor selection mechanism has inspired simple robust and distributed solutions for some well-specified problems ranging from different communication network routing designs^31^,^32^,^33^ to the management of heterogeneous network resources^34^ and the network selection for vehicular telematics^35^.

This investigation considers an attractor selection-inspired signal stage-switching mechanism for traffic intersections that goes beyond conventional signal control updating rules. The overall envisioned signalised traffic network is shown in Fig. 1, which depicts how to adapt the cell’s attractor selection mechanism to a signal control design. To be specific, we treat each signalised intersection as an individual cell and multiple networked intersections as a population of cells. The right of way on roads is indeed a kind of constrained spatiotemporal resource in a traffic network, which is expected to be utilised in an effective manner (i.e. appropriately allocated to different traffic flows associated with different movements arriving at or departing from each intersection) for the sake of alleviating traffic congestion and improving traffic efficiency. The traffic network can be regarded as the environment where the population of cells co-exists and co-evolves. As each intersection is conceptualised as a cell, the influence of the dynamics of one specific intersection on its neighbours is conceptualised as the indirect interaction among neighbouring cells sharing environmental resources. At this point, we can apply the mechanism (i.e. attractor selection) governing the adaptive dynamics of a cell’s gene network (i.e. switching between stable genetic programs to adapt to variations in the environment) to control each intersection, so that the intersection is able to accommodate the dynamic nature of the traffic network, and the global intersections can co-evolve in a distributed way to achieve a high traffic efficiency by autonomously and adaptively assigning the right of way on roads at each intersection, similar to the adaptive behaviour of cells. All details concerning the mathematical model formulating attractor selection as well as concerning our attractor selection-inspired signal-control algorithm are provided in the Methods section. Based on this, we illustrate the influence of the attractor selection-based control algorithm on the dynamics of the global traffic network and its potential and efficiency by testing it under different generic traffic conditions. Most importantly, the algorithm proposed here is simple, generic, self-adaptive and distributed, and may thus accelerate our process towards an intelligent and self-organised signalised traffic system.

## Results

In order to validate the bio-inspired signal-control algorithm proposed in this study, we carry out numerical simulations on two square lattice-type traffic networks of different sizes. As shown in Fig. 2, a simple network is set up that has only 2 × 2 = 4 intersections, all of which are boundary junctions. In addition, we also test the proposed mechanism on a large-scale network composed of 20 × 20 = 400 intersections. Each link of a network is assumed to have 2 movements; a through movement and a left-turn movement. For calculating the attractor selection model, we employ parameter settings similar to those used in the literature^30^: *P* = *C* = 0.01, 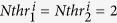 and 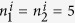 for ∀*i* (see the equation (10) in the Methods section). The time interval for updating the differential equations is fixed as Δ*τ* = 0.01 s. The total simulation time is set to 1.5 × 60 = 90 min, and the phase duration, i.e. the parameter Δ*δ*, is fixed at Δ*δ* = 25 s. For demonstration purposes, the minimum time headway of traffic flows, *TH*_*min*_, is assumed to be 1 s/veh, and the average flow speed, *avgSpeed*, is 45 km/h. Furthermore, we also assume that the length of all vehicles, *vehLen*, is 5 m, and that the length of any link connecting two adjacent intersections, 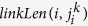, is 500 m. Then, a link travel time can be calculated by 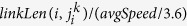 s. For simplicity, the averaged travel time over which a vehicle progresses from one movement at an intersection to another movement at a neighbouring intersection, 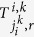, is set as the value obtained by multiplying 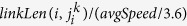 by a discount factor, *γ* = 0.6,


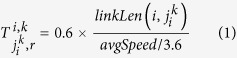


for ∀*i*. *k*, *r*, 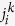.

**Figure 1:**
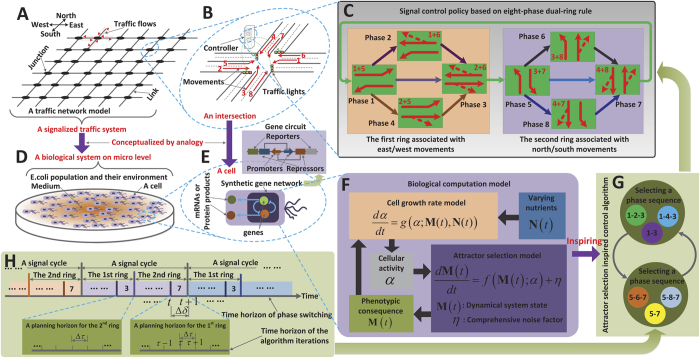
Signal control of a global traffic network inspired by biological adaptability on the cellular level. (**A**) A signalised traffic network modelled as a grid-type directed graph where a junction denotes a typical signalised intersection with four legs and a link represents a road section with two lanes per direction. (**B**) Details of a typical signalised intersection, which is responsible for organising eight traffic movements by operating different signal lights in different directions. These signal lights are controlled by a local controller, and the movements are numbered according to the convention of the NEMA (National Electrical Manufacturers Association). (**C**) A typical eight-phase dual-ring signal control policy in which each ring manages a group of four phases corresponding to four movements (two through movements and two left-turn movements) and their possible right-turn ones. (**D**) *E. coli* cells co-existing in a nutrient environment. (**E**) An *E. coli* cell whose gene network is modelled by a gene circuit with two mutually inhibitory operons, each of which is composed of a repressor, a constitutive promoter and a reporter. (**F**) The mathematical model system capturing the dynamics of cellular adaptive response generated by the gene network to environmental changes, which is mainly composed of a cellular growth rate model and an attractor selection model. These models are employed to design an autonomous and adaptive algorithm for dynamically adapting phase-switching of the signal control policy in (**A**) to the traffic dynamics. (**G**) The phase transition corresponding to the eight-phase dual-ring signal-control policy. (**H**) The time horizons for phase-switching and for an iteratively running control algorithm.

In the traffic network simulations, we assume that the signal phase at each intersection initially stays at a value randomly selected from eight alternatives (See Fig. 1(C)), and that vehicle queues on all approaches of each internal intersection are randomly, independently and uniformly generated from the interval [0, 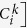 as initial conditions (the calculation of the averaged capacity, 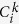, is referred to in the Methods section). The initial vehicle queues on the external movements of the network are set to zero. During the simulations, we generate the arrivals of vehicles (as inputs of the traffic network) from the exterior of the traffic network by following a Poisson distribution characterised by the arrival rate of *λ* = 300 (veh/h/movement). We further consider two general cases in our simulations: (i) all the movements at any intersection experience uniform traffic demand, i.e. the ratio of the traffic demand on the through movements to that on the left-turn is set to 1:1 in this simulation case, and (ii) the traffic demand on the through movements overweighs that on left-turn movements by a ratio of 3:1, which indicates that the arrival rate of the through movement of an external link is set to 2*λ* × 3/(3 + 1), while that of the left-turn movement of the same link is 2*λ* × 1/(3 + 1) in this case. The turning fraction of vehicles that travel from a movement *r* at intersection 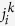 to a movement *k* at adjacent intersection *i*, 
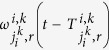
, is fixed at 1/(1 + 1) = 0.5 for ∀*i*, *k*, *r*, 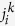 in the first case. On the other hand, the second case is set as follows:


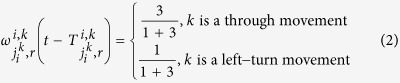


**Figure 2:**
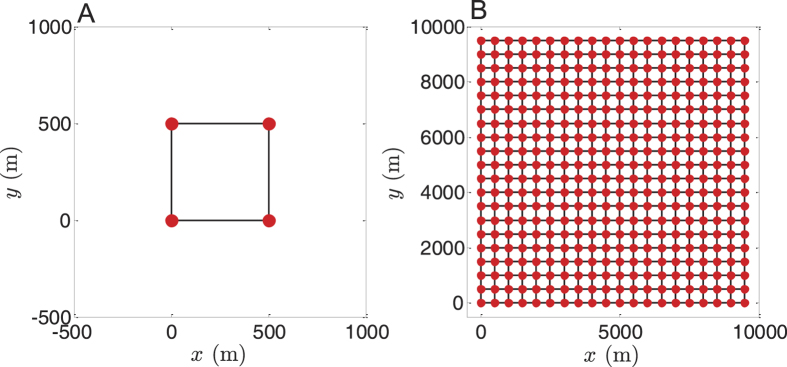
Two square lattice-type traffic networks on which simulation experiments are carried out. (**A**) A small network with 2 × 2 junctions. (**B**) A large network with 20 × 20 junctions.

**Figure 3:**
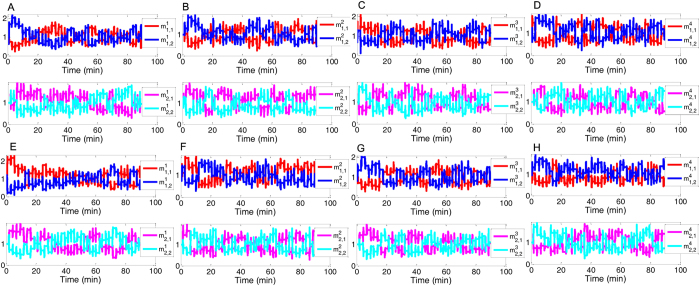
Dynamics of the decision variables associated with each intersection during simulation of the 2 × 2 traffic network under two different cases. (**A–D**) show the time-variations of the decision variables 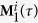 and 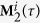 of the top-left, bottom-left, top-right, and bottom-right intersections in the small network given in Fig. 2(A), respectively, under the first case condition. Here the ratio between the through traffic demand and the left-turn traffic demand is =1:1. (**E**–**H**) display the results of the counterparts under the second case condition, where the ratio between the through traffic demand and the left-turn traffic demand is =3:1.

We first examine how intersections driven by the attractor selection mechanism adjust their signal control adaptively to traffic network dynamics by performing simulations on the simple traffic network. As shown in Fig. 3, the time-variations of the decision variables 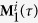 and 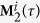 of any intersection are different from one another, which implies that the signal control modes among these intersections differ as well. Furthermore, during different time periods, the signal phase sequence of each control ring selected by each intersection changes dynamically according to the dynamics of the traffic condition of their legs. Indeed, it can be confirmed from Fig. 3 that two types of stable attractors exist during most of the simulation time at any intersection, one of which has a higher level of 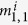 (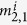) but a lower level of 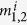 (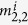) while the other is on the contrary associated with a lower 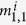 (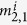) and a higher 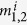 (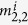). At some time periods, another attractor is also selected under which both of the decision variables are approximately equal. Also, it can be observed that any intersection driven by the attractor selection mechanism cannot stay at the same attractor for long. Instead, an intersection always switches among different attractors, which means that it selects different phase-switching sequence solutions in real time according to changes in its external environment (the varying traffic condition of the relevant movement is simulated by the state equation (4) in the Methods section). For example, as shown in Fig. 3(A), which illustrates the dynamics of the attractor selection of the first (top-left) intersection in Fig. 2(A) in the first simulation case, this intersection initially goes into a regime with two attractors, where the attractor with a larger 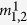 is selected for the first control ring. From about 0 to 10 min, this intersection stays at this attractor, which suggests that it chooses the corresponding phase-switching sequence, 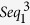, for the first control ring during this time period. Then, it switches to a new attractor with approximately equal 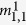 and 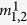 at about 10 min such that the second phase-switching solution, 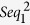, is selected. However, it immediately returns to the previous attractor with larger 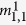 at about 12 min, deciding on 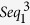 again for the first ring due to the fluctuation in the traffic flow. During the period of [12, 20] min, the intersection returns to select the second phase-switching solution, 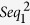, for the first ring due to the selection of the attractor with 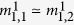. Conversely, for the second control ring, the phase sequence 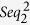 is chosen during about the first 5 min since the attractor with approximately equal values of 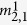 and 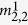 is selected. However, after this time, the intersection switches from this attractor to another one with a higher level of 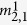 and stays at the new attractor for about 5 to 20 min, during which time it employs the phase-switching sequence 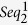 for the second ring. A similar behaviour of switching between attractors can also be found after 20 min, and at other intersections (see Fig. 3(B–D)) or other simulation cases (See Fig. 3(E–H)). This behaviour, by which the phase-switching is induced by the attractor selection and is adapted to the varying traffic on the roads in real time, is similar to the adaptive response of a cellular gene network to environmental changes.

**Figure 4:**
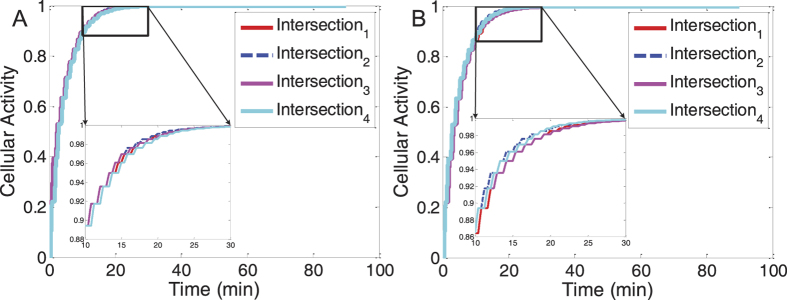
The time-variation of the cellular activity of each intersection during the simulation of the small network. (**A**,**B**) Show the time-varying cellular activity of each intersection of the small network in the first (the through traffic demand: left-turn traffic demand =1:1) and second (the through traffic demand: the left-turn traffic demand =3:1) simulation cases, respectively. The labels, Intersection_1_, Intersection_2_, Intersection_3_, Intersection_4_ correspond to the top-left, bottom-left, top-right and bottom-right intersections shown in Fig. 2(A).

Recalling equations (12), (13) and (15) from the Methods section, it can be seen that the cellular activity, i.e. the parameter *α*^*i*^(*τ*), comprehensively reflects the goodness of a cell (the intersection *i*) which is related to the dynamics of the external nutrients (the road resources), 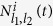 (*l*_1_, *l*_2_ = 1, 2), and the level of the gene expressions (the decision variables), 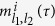 in real time. Specifically, a longer vehicle queue on a movement *k* of the intersection *i*, i.e. a higher level of 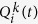 implying potential traffic congestion, can lead to a lower level of the utility parameter 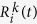 (see equation (12)), which turns out to reduce the corresponding 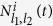 (see equation (13)). Without sufficient nutrients (i.e. with lower levels of 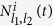 resulting from higher levels of the queue state 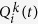), the cellular activity *α*^*i*^(*τ*) cannot be high unless appropriate gene-expression programming (an adaptive attractor represented by a specific 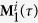 or a specific 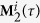) is selected to compensate for the decrease in the nutrients. At this point, the cellular activity parameter, *α*^*i*^(*τ*), actually indicates the fitness degree of an intersection in a changing traffic environment, so we examine the time-variation of *α*^*i*^(*τ*) for ∀*i* under the conditions of both simulation cases. Figure 4 illustrates the convergence of the cellular activity of each intersection over the simulation time. From the results shown in Fig. 4(A,B), we find that the cellular activities of all the intersections driven by attractor selection can eventually converge to 1 at the steady state, no matter whether the traffic demand on the through movement dominates that on the left-turn movement. This means that the overall network is able to accommodate different kinds of traffic loads by dynamically adjusting its signal controls. Interestingly, comparing the time-variations of different intersections’ cellular activities, it is obvious that the differences among the time-convergence curves of these cellular activities obtained in both simulation cases are slight. The reason behind this result lies in the fact that local neighbouring intersections, induced by the attractor selection mechanism, control their signals in a cooperative manner through indirect interaction by sharing road resources among them. To be specific, the signal operation of an intersection upstream, which directly determines the throughput of this intersection, has a great influence upon the traffic flow downstream. As the signal control of a neighbour downstream essentially depends on the dynamics of the traffic flow, the selection of an appropriate phase-switching sequence is indirectly influenced by the output of the upstream intersection, namely, by its signal-control. By introducing biological adaptability, multiple intersections, like cells coexisting, dynamically regulate their signal policies to allow their activities to increase as much as possible. In this way, the intersections can co-evolve to eventually achieve a high fitness.

**Figure 5:**
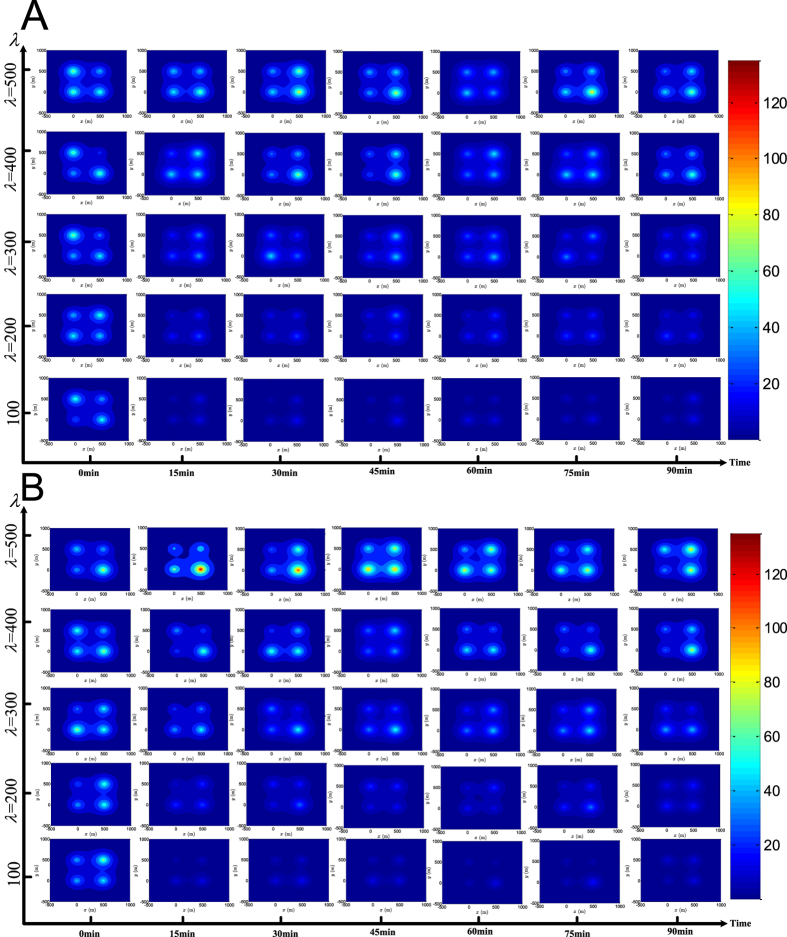
Evolution of the small-scale traffic network with the bio-inspired signal control based on attractor selection. (**A**,**B**) Illustrate the results captured in the first and the second simulation cases, respectively. The degree of the colour on these maps indicates the level of the pre-defined congestion index, *β*(*p*, *t*).

**Figure 6:**
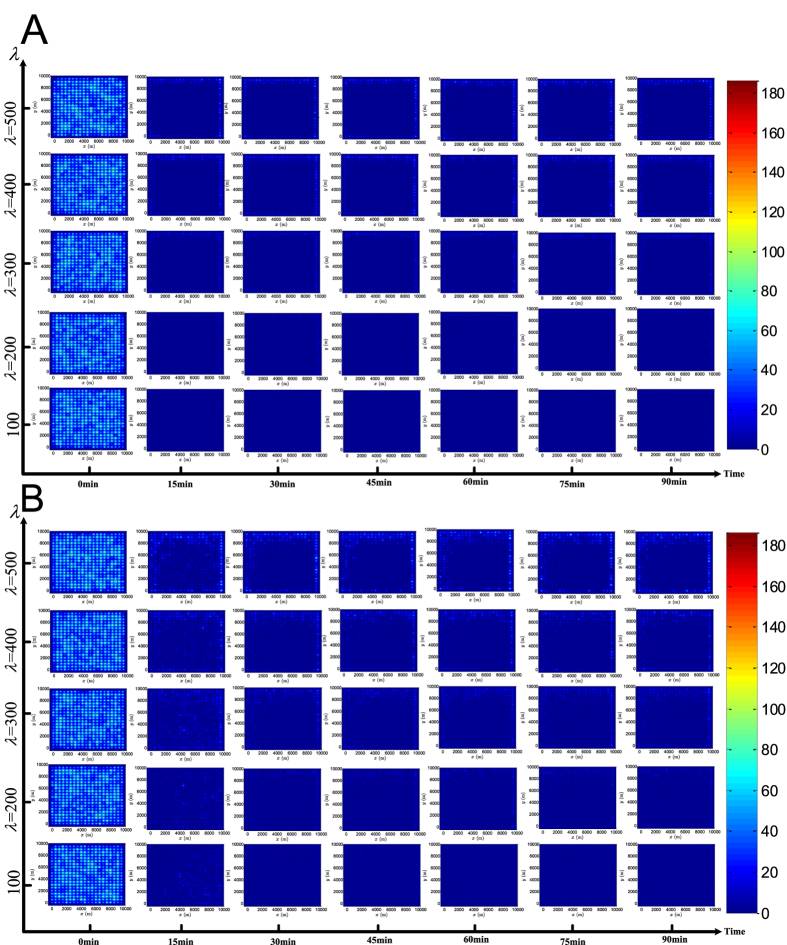
Evolution of the large-scale traffic network with the bio-inspired signal control based on attractor selection. (**A**,**B**) Are the results obtained in the first and second simulation cases, respectively. The degree of the colour on these maps represents the level of the pre-defined congestion index, *β*(*p*, *t*).

To gain a better understanding of the effect of the cellular attractor selection on the evolution of the overall traffic network, we test the bio-inspired signal control under both the small and large-scale traffic networks with different vehicle arrival conditions, where the parameter *λ* is varied from 100 to 500 (veh/h/movement). To show the evolution process, we first introduce a spatiotemporal metric, *β*(*P*, *t*), where *P* denotes the position vector of a geometric location in the traffic network, and *t* is the time index, named ‘congestion index’, to represent the spatiotemporal traffic state of the global network area. *β*(*P*, *t*) is defined as follows:


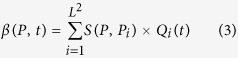


where *Q*_*i*_(*t*) is used to quantify the impact arising from the total vehicle queues formed at the intersection *i* at *t* on any other location in the network and is defined as 
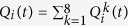
. *S*(*P*, *P*_*i*_) quantifies the geographical factor that causes the impact of the intersection *i* on the location *P* to fade. *S*(*P*, *P*_*i*_) can be simply defined as a monotonically-decreasing function of the relative distance between the locations *P* and *P*_*i*_. For the sake of demonstration, we use a negative exponential function to formulate such an *S*(*P*, *P*_*i*_): *S*(*P*, *P*_*i*_) = exp(−3.8 ‖*P* − *P*_*i*_‖/*linkLen*), where ‖·‖ denotes the Euclidean norm and *linkLen* is set to 500 m. It can be seen that *S*(*P*, *P*_*i*_) ranges within (0, 1] and decreases with increasing relative distance, ‖*P* − *P*_*i*_‖. Based on *β*(*P*, *t*), we plot the spatiotemporal state of the overall traffic network through a series of snapshots of the small and large-scale traffic networks captured every 15 min during simulations, as illustrated in Figs 5 and 6, respectively. As the simulations are performed with independent random initialisations, both Figs 5 and 6 show that the traffic state of any intersection in the coloured map captured at the initial time instant, *t* = 0 min, is different from that of all other intersections. Compared with other locations, the levels of *β*(*P*, *t*) achieved at the intersections are commonly higher than those of other places in the same network during simulations, which shows that some intersections are actually traffic hotspots. More importantly, even with independent and different initialisations, either small- or large-scale traffic networks induced by attractor selection-based signal control can commonly evolve over time to a state where relatively low levels of *β*(*P*, *t*), i.e. a light traffic load, are achieved by the global network. This indicates that the overall traffic pressure on the network faced at the beginning can gradually be relieved later by employing the proposed signal control. Furthermore, the snapshots of the traffic state of the network with different sizes and different vehicle arrival rates captured at a later simulation stage, such as at *t* = 60, 75 *or* 90 min, show that the deviations among the levels of *β*(*P*, *t*) achieved at different intersections are gradually diminished when compared to the initial maps at *t* = 0 min. The phenomenon is more obvious with the light and medium vehicle arrival rates *λ* = 100, 200 *or* 300 veh/h/movement. We also find that, with the higher traffic demands obtained by setting *λ* = 400 *or* 500 veh/h/movement, some intersections (such as those at the top-right and bottom-right in the maps captured at *t* = 30 *and* 45 min under *λ* = 400 veh/h/movement and at *t* = 30, 45 *and* 75 min under *λ* = 500 veh/h/movement shown in Fig. 5(A), as well as the same bottom-right intersection in the maps at *t* = 15 *and* 30 min under *λ* = 500 veh/h/movement shown in Fig. 5(B)) may suffer from an increased *β*(*P*, *t*) by chance. This is mainly caused by the dynamic nature of the traffic flow merging at the intersections and the randomness in the vehicle arrival time. Nevertheless, these occasional heavier traffic hotspots can still be eased to some extent at the final stage at *t* = 90 min as demonstrated in Fig. 5(A,B).

By comparing the results obtained in the first simulation case (see Fig. 5(A)) with those obtained in the second (see Fig. 5(B)), especially under the settings *λ* = 400 *or* 500 veh/h/movement, it can be found that the overall traffic states in a series of maps captured in the second case are slightly heavier than those captured in the first case. This phenomenon can also be confirmed in the simulations of the large-scale network, as shown in Fig. 6. In fact, since the through traffic demand is set in the second simulation case to absolutely dominate the left-turn traffic in the first case by a ratio of 3:1, the imbalance in the traffic demand can have a certain influence on the dynamics of the traffic network, as well as on the phase-switching in the signal-control. But such unbalanced traffic demand does not always lead to a significant degradation in the overall performance of the network. As shown in Fig. 5(B), the congestion index achieved at the traffic hotspots (the intersections) at *t* = 90 min is lower than 90 veh (i.e. below 11.25 veh per movement) on average, and when comparing both (**A**) and (**B**) in Fig. 5, the difference between the impacts arising from the balanced and the unbalanced traffic demand on the network evolution is much gentler under settings of *λ* = 100, 200 *or* 300 veh/h/movement. Also, the levels of the congestion index achieved at different places including these intersections and other locations in the traffic area are approximately uniform at the later evolution stages *t* = 60, 75 *or* 90 min as shown in the maps with *λ* = 100, 200 *or* 300. This means that the proposed signal control based on attractor selection is able to balance the overall traffic load of the network to some degree, regardless of the unbalanced traffic demand realised.

We further point out that the aforementioned facts are also confirmed by examining the results obtained from the large-scale network given in Fig. 6. By comparing the results of both Figs 5 and 6, it is further found that the congestion indices of the boundary intersections (indeed, all of the intersections in the 2 × 2 network in Fig. 5 are boundary intersections) are slightly heavier (marked by a hotter colour) than those of the internal intersections. This phenomenon is more evident under *λ* = 400 *or* 500 veh/h/movement in these figures. This finding is easy to understand when recalling that some approaches of the boundary intersections are external, and the number of vehicles arriving on the external approaches increases along with arrival rate *λ*. Additionally, it is interesting to see that the levels of the congestion indices achieved by the large-scale network at the later evolution stages (*t* = 60, 75 *and* 90 min) are lower on average than those achieved by the small-scale network, and the congestion indices at different places are more uniformly distributed on the maps of the large-scale network. Logically, the large-scale network has a total of 20 × 20 = 400 intersections, much more than the number (2 × 2 = 4) in the small-scale network. This also implies that potential spatial resources in the large-scale network are much greater. Specifically, there are (20 − 1) × 20 × 2 = 760 inter-connecting links and 4 × 2 + (20 − 2) × 4 = 80 input traffic streams in the large-scale network (note that these input flows are associated with the boundary intersections of which the large-scale network has 4 + (20 − 2) × 4 = 76, with four of them being at the four corners and the others arrayed at the four edges of the grid), while the 2 × 2 network has only 4 interconnections and 8 input streams in total (see Fig. 2(A,B)). Thus, in terms of the ratio of the spatial capacity provided by the network area over the traffic demand, i.e. (760/80) ≫ (4/8), the potential traffic capacity of the large-scale network is greater than that of the small-scale network. This allows the overall traffic load to be more uniformly distributed over more interconnections (more space) by the large-scale network with the proposed signal control, and thus we see a series of cooler maps captured during the large-scale network evolution. In summary, from the maps shown in Figs 5 and 6, the evolution of the network to the traffic state with a low congestion index *β*(*P*, *t*) on average confirms that the adaptability induced by the cellular attractor selection, as a kind of biological intelligence, can be appropriately applied to inspire the design of intelligent signal control in order to alleviate traffic congestion and balance the distribution of the traffic load imposed on the network.

**Figure 7:**
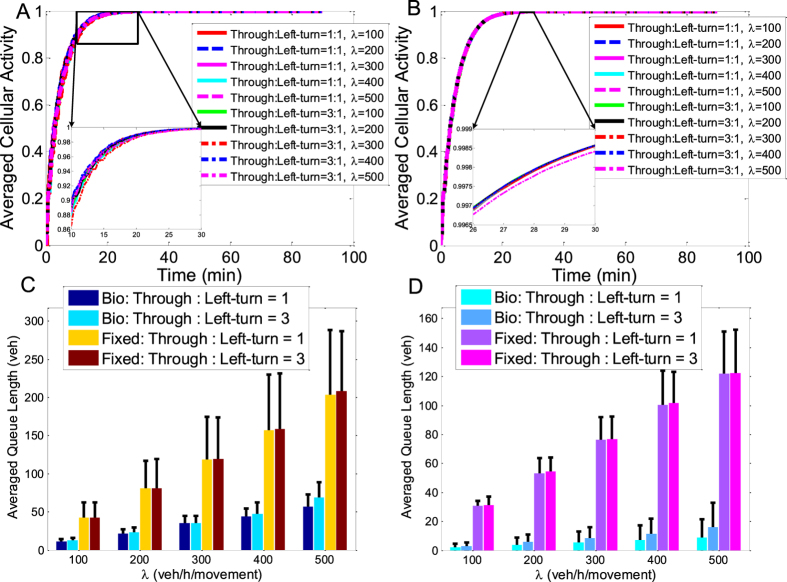
Time variation of the averaged cellular activity of the overall traffic network and the average and standard deviations of the overall vehicle queue length. (**A,B**) Illustrate the convergence of the averaged cellular activity of the overall traffic network with different settings over simulation time under the condition of the small- and large-scale networks, respectively. The results corresponding to different simulation settings are marked by different types of curves. (**C,D**) Give the averaged queue lengths obtained with different simulation settings in the small and the large networks, respectively, which are represented by bar charts, and also include the corresponding standard deviations that are denoted by a series of error bars. Specifically, the worst case of the queue length of the overall network can be recognized by the upper end of the error bar. In (**C,D**), the results of the proposed bio-inspired control scheme are marked by Bio, while those of the fixed-time control are marked by Fixed.

Next, we examine the time-variation of the cellular activity of the overall network, as well as the averaged vehicle queue length during the preceding 30 min, under different vehicle arrival rates and traffic demands. We also carry out comparative experiments on a conventional signal control scheme, i.e., the widely adopted fixed-time control where the phase sequence solution and the signal cycle are pre-specified and fixed. Specifically, in the initialisation of the fixed-time control, each intersection randomly and independently selects a specific phase sequence for the first and for the second control rings according to the NEMA principle (See Fig. 1(C)), respectively, and implement the pre-set phase sequence scheme throughout the whole simulation period. For comparison, the fixed-time control is tested under the same traffic conditions as those of the proposed bio-inspired scheme. On one side, to analyse the evolution of the cellular activity defined in the attractor selection model, we derive the averaged cellular activity of the overall traffic network at each time instant by averaging all the intersections cellular activities at the same time instant, and then we plot the averaged cellular activity over the whole time, as shown in Fig. 7(A,B). On the other side, the performance of the proposed control is also compared with that of the fixed-time control in term of the basic metric, i.e., the averaged vehicle queue length. To obtain the averaged vehicle queue length and the corresponding standard deviation, we first average the total vehicle queue of each intersection, *Q*_*i*_(*t*), over the later simulation stage whose duration is from 60 to 90 min, and then calculate the mean queue length of the overall network by averaging the time averages of all the intersections. Subsequently, the standard deviation can be derived as the variance among the time averages of all the intersections. The comparative results are shown in Fig. 7(C,D).

From both (**A**) and (**B**) in Fig. 7, it can be found that, with bio-inspired signal control based on cellular attractor selection, the signal phase-switching sequences are adaptively selected to boost the cellular activity (fitness) of the global intersections, enabling the overall traffic system to accommodate different traffic conditions (vehicle arrival rates and traffic demands) and network sizes. This confirms the similar findings presented in Fig. 4. More significantly, regardless of the different simulation settings and random and independent initialisations, the cellular activity of the overall networks of small and large sizes consistently converges to 1, meaning that the robustness of the signal control performance over the traffic networks is promoted by cellular attractor selection. That is, the signalised traffic network can be induced to evolve towards a robust and adaptive system by characterising each individual intersection that incorporates biological intelligence into its signal control. The potential for cellular attractor selection to boost the adaptability and robustness can also be understood by comparison between the results obtained in the small- and large-scale networks. As can be seen from Fig. 7(B), differences between the results corresponding to different simulation settings obtained in the large-scale network are less obvious than those in the small-scale network shown in Fig. 7(A). The phenomenon shown here further confirms the observation from Fig. 6 that a larger spatial capacity can be exploited to organise the traffic demand, and that the traffic load can be more uniformly allocated on the interconnections over the large-scale network by cellular attractor selection. Also, by focusing on the results of the bio-inspired scheme in Fig. 7(C,D) (See the bars marked by ‘Bio’), it can be found that a larger vehicle arrival rate logically increases the averaged vehicle queue of the overall network since it boosts the input traffic volume, and that the imbalance of traffic demand over the network impacts on the formation of vehicle queues at intersections, slightly increasing the vehicle queue lengths. The similar trend can also be observed in the figures obtained by applying the conventional fixed-time control (See the bars marked by ‘Fixed’). However, when the proposed bio-inspired signal control is used, the upper bounds of these vehicle queue lengths, representing the worst results obtained at the later simulation stage, are all less than 100 veh on average. More importantly, in the small-scale traffic network simulation situation where the through traffic demand and the left-turn are set to be equal, i.e., ‘Through:Left-turn=1’, the bio-inspired scheme shows about a 72.64% decrease of the averaged vehicle queue length on average as compared to the fixed-time control. Besides, in the situation where the small-scale traffic network is simulated under the imbalanced traffic demand, i.e., under ‘Through:Left-turn=3’, the averaged vehicle queue length obtained by the bio-inspired scheme is about 70.18% lower than the result of the fixed-time control. As expected, in the large-scale traffic network simulation, the averaged vehicle queue length of the bio-inspired signal control is only about 6.54% of the fixed-time control on average under the ‘Through:Left-turn=1’ condition, and about 10.69% of that of the fixed-time control on average under ‘Through:Left-turn=3’. The figures presented above, comprehensively reveal the strength of this appealing and promising mechanism that is intrinsic in biological behaviour as result of very long-term natural evolution. This is consistent with the conclusions on the advantages of cellular attractor selection from previous investigations^34^,^35^, and supports an argument from the literature^30^ on the topic of the potential of this type of biological intelligence to facilitate the development of an artificial system capable of robustly and adaptively responding to uncertainties and changes in the external environment, where centralised control, large-scale sensors and transducers are not necessary.

## Discussion

In this work, we have explored the influence of cellular attractor selection-based signal control on the dynamics of a traffic network. Following the biological inspiration for engineering designs, we incorporated the biological mechanism behind attractor selection into the strategy of selecting the appropriate signal phase switching sequences for each control ring, and induced all individual intersections to regulate their signal controls in a fully distributed, autonomous and adaptive manner like cells co-existing and co-evolving, enabling the overall signalised network to accommodate the dynamic nature of the traffic network in real time. By conducting numerical simulations with different parameter settings for the network size, vehicle arrival rate and traffic demand, and capturing a series of snapshots on the dynamics of the traffic network, we illustrated that networks on both small and large scales can be driven by cellular attractor selection to evolve towards the traffic state with a high degree of fitness, where the overall congestion index of the traffic hotspots and other locations in the network is achieved at a relatively low level on average when compared to that at the initial stage. As a low congestion index can imply a good traffic service level, the bio-inspired signal-control mechanism was validated to have the capacity of promoting traffic flow management at a high service level. In particular, we have demonstrated that, even under different traffic conditions such as increased vehicle arrival rates and unbalanced traffic demand existing in both types of traffic networks, the overall cellular activity of the network, indicating the fitness of the global intersections, can always gradually converge to 1 along with the network evolution over time, and that the vehicle queue of the overall network formed at the later simulation stage on average, or even the upper bound of the vehicle queue length obtained in the worst case, can always be controlled below the minimum service capacity of a signal cycle of an intersection by adopting such cellular attractor selection-inspired signal control, regardless of the increasing arrival rates and of the imbalance in traffic demand. Thus, we can conclude that cellular attractor selection can induce adaptive and robust signal control in the traffic network. Additionally, we have also found that bio-inspired signal control may be more suitable and promising when employed in a large-scale traffic network. Comparative simulation results have shown that, with the bio-inspired control scheme, traffic load can be more uniformly allocated on the large-scale network where a large number of interconnections indicate more spatial resources that can be exploited to ease the traffic pressure by the bio-characterised intersections. In conclusion, we have gained a deep insight into the potential of cellular attractor selection applied in inspiration for an autonomous, adaptive and robust signal control design for a traffic network, showed that such a bio-inspired paradigm is feasible for managing large-scale traffic flows and confirmed its promotion of the high fitness of the overall traffic network evolving over time.

As the first step in adapting the cellular attractor selection to signal control in a traffic network, this investigation has shed light on how this system impacts on traffic dynamics from the standpoint of a road network, and presents a promising, bio-inspired solution framework of high scalability for signal control design applied in large-scale traffic networks with the goal of promoting intelligent traffic organisation and management in urban areas. Nevertheless, further study related to the development of biologically inspired signal control remains to be conducted in the future. It would make sense to provide an integrated approach, which combines the bio-inspired solution proposed with other information technologies currently employed in transport systems such as connected vehicles and mobile wireless sensor networks, such that the comprehensive performance of the bio-inspired signal control can be enhanced with the assistance of richer, more real-time and more accurate traffic information collected by other advanced information systems.

## Methods

The methods involved in this study are essentially composed of two parts, one which mathematically models the flow dynamics for a traffic network, and another which elaborates on the bio-inspired computational model employed, i.e. the attractor selection model, as well as the inspiration from this model to the design of a bio-inspired signal control algorithm. All of the numerical simulations are conducted in MATLAB with object-oriented programming.

### Modelling traffic flow dynamics

Without loss of generality, we consider in this study a typical traffic network as an *L* × *L* square lattice, as shown in Fig. 1(A), where the total number of signalised intersection is *L*^2^. Thus, the current vehicle queue, 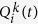, in the movement *k*(*k* = 1, 2, …, 8) at a four-way signalised intersection *i*(*i* = 1, 2, …, *L*^2^), as given in Fig. 1(B), can be formulated by a discrete-time state equation using the principle of flow conservation:


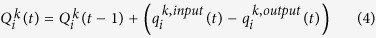


where *t* is the discrete time step. 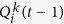 represents the previous vehicle queue at the instan *t*−1. We assume that the time interval for sampling the traffic flow state is Δ*δ*, i.e. the duration between the time instant *t* − 1 and *t*. 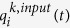 and 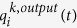 are the inputting and outputting flows associated with the movement *k* of the intersection *i* during [*t* − 1, *t*), respectively. For simplicity, we represent the state of an intersection as 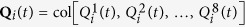, which collects the current vehicle queues in all the movements of this intersection *i*. Here, col[·] denotes a column vector.

Furthermore, we denote the minimum headway time between vehicles in a queue by *TH*_*min*_ (sec/veh) and the current signal control as a binary variable, 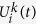. We set 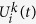 to 1 if the movement, *k*, has the right of way with the green light on, and to 0 if the red light is on. Then, the signal control of the intersection *i* at the time instant *t* can be represented as 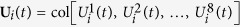. Thus, we can calculate 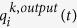 by


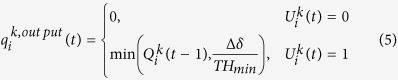


for ∀*i* and ∀*k*.

In general, the intersection *i* can be an interior junction surrounded by four adjacent intersections, or it can be a boundary junction of the lattice-type network so that *i* only has two adjacent intersections. Therefore, in order to calculate 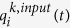, two possible situations should be recognised: (i) this movement *k* is an internal movement inside the traffic network, where vehicles come from a neighbouring intersection and go to the intersection *i* (namely, *k* connects the intersection *i* downstream and an adjacent junction upstream); and (ii) *k* is an external movement, where the vehicles come from the outside of the traffic network. Therefore, for the situation (i), we denote an intersection that is next to the intersection *i* and upstream of the movement *k* as 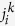 and represent the set of movements where the vehicles will go from the intersection 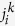 to *i* as 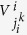. Then, we can derive 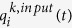 as


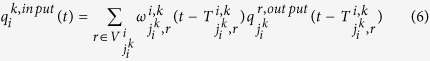


where 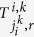 denotes the travel time on average when a sub-flow in the movement *r* travels from the intersection 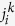 into the movement *k* of the intersection *i*, which should be rounded to an integer, and 
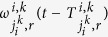
 denotes the ratio of the number of vehicles in such a sub-flow over 
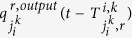
 at the time instant 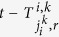.

In situation (ii), following well-established methods from the literature, we consider a situation where the arrival of vehicles from external movements, i.e. the input of the traffic network, follows a Poisson distribution with an averaged arrival rate *λ* (veh/sec). Thus, when a movement *k* is external, we can simply calculate its 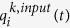 by


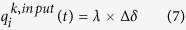


Combining equations (4), (5), (6) and (7) yields a discrete-time state model for the traffic network dynamics, with the state of the network being 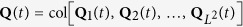, controlled by the real-time signal control:


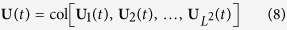


### Adapting the Attractor Selection Model to Signal Control

The dynamics of *E. colis* attractor selection can be mathematically formulated by a group of stochastic nonlinear differential equations shown in (9)^30^, which are developed on the basis of a genetic toggle switch model:^36^


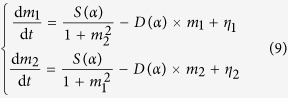


Accordingly, the state of an *E. coli* cell, i.e. its metabolic phenotype, is represented by a pair of time-dependent variables (*m*_1_, *m*_2_), each of which indicates an mRNA concentration or its relevant protein product. Equations (9) are a two-dimensional nonlinear dynamic systems whose potential space has two stable states, i.e. two attractors, where one of the two variables dramatically overtakes the other: *m*_1_ ≫ *m*_2_ or *m*_1_ ≪ *m*_2_. The parameter *α*, called *cellular activity*, always ranging within [0, 1] and is used to reflect the growth rate of the cell. *η*_1_ and *η*_2_ are two independent white Gaussian noises that define certain fluctuations in the gene expressions caused by internal and external factors. In the attractor selection model, the functions *S*(*α*) and *D*(*α*) denote the rates of nutrient synthesis and decomposition, respectively, which depend on the cellular activity *α* and can be formulated as two monotonically increasing functions: *S*(*α*) = 6*α*/(2 + *α*) and *D*(*α*) = *α* according to the literature^30^. Importantly, *α* is a result of the interaction between the cell and its environment, implying that it can be modelled as a function of the cellular phenotypic consequences regulated by its gene expressions, (*m*_1_, *m*_2_), and the nutrient conditions, which is expressed by a typical cell growth rate model given as^37^


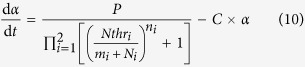


where the parameters *P* and *C* denote the rates of producing and consuming *α*, respectively. *Nthr*_*i*_(*i* = 1, 2) is the threshold corresponding to the nutrient *i* to produce *α*, while *n*_*i*_ is the relevant sensitivity. The variables (*N*_1_, *N*_2_) represent the levels of the two nutrients which are supplemented by the external environment as well as being supplied by enzyme expressions encoded by the corresponding operons in the cellular gene network.

From Equations (9) and (10), we can understand that the adaptive attractor of a *E. coli* cell is allowed by the synergism between its bistable gene expression dynamics, mathematically represented by (*m*_1_, *m*_2_), the dynamics of the environmental conditions represented by (*N*_1_, *N*_2_), and the stochastic fluctuations denoted by (*η*_1_, *η*_2_). The cellular activity *α*, treated as the cellular fitness in the environment, is the outcome of the interaction between the cells current state, i.e., (*m*_1_, *m*_2_), and the environmental nutrients, i.e., (*N*_1_, *N*_2_). *E. coli* cells are inherently able to select a more suitable gene expression state, i.e., an adaptive attractor, to adaptively respond to changing environmental conditions, which in turn allows for higher cellular activity, i.e., increasing cellular fitness, without assistance of a signal transduction pathway. This is also called cellular adaptive attractor selection, as stated in the work of Kashiwagi *et al.*^30^. In order to employ such biological mechanism to realize the design of an adaptive signal control system, three key issues are appropriately addressed as the following, which include: (i) *modeling the environmental conditions*, i.e., providing a formation of the nutrients in the context of traffic system; (ii) *matching attractors with possible signal control solutions*, i.e., linking the state space of the dynamical system represented by the attractor selection model to the solution space of signal control and (iii) *formulating the cellular activity as an indicator of goodness of current signal control solution at an intersection*, such that this cellular activity can reflect the fitness of a cell (an intersection) in the dynamically changing environment.

Firstly, following the identification of an analogy between a signalised traffic system in Fig. 1(A) and a microbiological system in Fig. 1(D), we characterise the dynamics of the conceptualised ‘nutrients’ in the traffic environment and then lump such dynamics into the variables (*N*_1_, *N*_2_). Denoting the length of a vehicle on average as *vehLen* (m) and the mean speed of the traffic flow in a link connecting any two adjacent intersections as *avgSpeed* (km/h), we can calculate the averaged flow density of a lane associated with a movement, *avgDen* (veh/m/movement) as


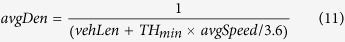


Subsequently, the averaged capacity of the lane corresponding to a movement *k* of the intersection *i* can also be derived using (11): 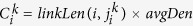 (veh/movement), where 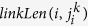 denotes the length of the link connecting the adjacent intersections *i* and 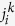. Then, we define the available traffic resources of an intersection as the “nutrients” available to a cell. The available resources are indeed the spatial resources of a lane in the traffic network, which are parameterised as a function of the length of the vehicle queue and the averaged capacity of the lane. We employ a sigmoid-type monotonically decreasing function to formulate the available resources, denoted as a parameter 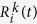, so that it can be regulated within [0, 1]:


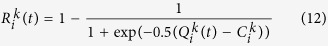


Following (12), we can further calculate the available resources of each leg of the intersection. Let us associate the mathematical notations 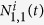, 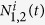, 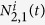 and 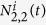 with the east, west, south and north legs of the intersection *i*, respectively. According to the specific labels of the movements in each leg as shown in Fig. 1(B), the parameters 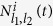 (*l*_1_, *l*_2_ = 1, 2) can be defined by calculating


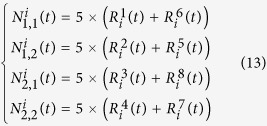


where 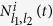 (*l*_1_, *l*_2_ = 1, 2) are treated as the time-varying environmental nutrients of the cell (the intersection *i*). From (12) and (13), it can be found that increasing the emerging vehicle queue 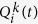 will decrease the parameter 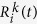, which leads to decreasing the nutrient parameters 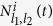. The more vehicles are waiting at the intersection, the higher the road occupancy is, and the fewer the residual road resources become, which indicates that the nutrients available to a cell are fewer. Besides, we point out that since the parameter 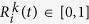, 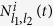 ranges within [0, 10], which is in accordance with the changing culture conditions exploited in a simulation experiment in the literature^30^.

Next, we employ a typical signal control design framework according to the NEMA convention as given in Fig. 1(C). Accordingly, three alternative phase-switching sequences are grouped in each ring of signal control. Specifically, for any signalised intersection (as illustrated in Fig. 1(B)), the first ring is associated with the east and west movements, and these movements can be operated in four different phases (1, 2, 3 and 4). There are three combinations of these phases, which correspond to three alternative phase sequences, namely 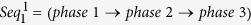, 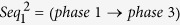 and 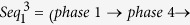

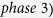
. Similarly, in the second ring, the alternatives are 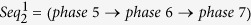, 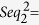

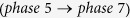
 and 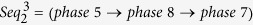. Such phase sequences of each ring can be combined in different ways to realise a control cycle, and there are 3 × 3 possible combinations. The essential step here to enable the intersection to be adaptive is to drive its controller to autonomously and dynamically select an appropriate phase switching sequence for each control ring from the alternatives according to traffic dynamics of the network. We introduce two decision vectors, 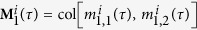 and 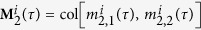, where 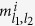 (*l*_1_, *l*_2_ = 1, 2) is the *l*_2_-th decision variable corresponding to the *l*_1_ control ring of the intersection *i* at the time instant *τ*, and treat these decision vectors as the biological parameters representing the metabolic phenotypes (gene expressions) of the cell. Then, we characterise the selection of these phase sequence solutions aforementioned as the cellular attractor selection. That is, possible signal control solutions are defined as attractors of the dynamical system represented by the attractor selection model (9). Compared with the second phase sequence solution 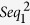, the phase sequence solutions 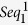 and 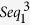 allocate more green time (an additional phase 2 or 4) for the eastern movements (1, 6) and for the western movements (2, 5), respectively. 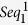 or 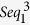 should be appropriately employed for the first control ring when the traffic flows in the associated movements overweigh those in the opposite direction. Similarly, compared with 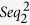, 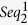 and 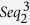 allocate more green time (an additional phase 6 or 8) for the southern movements (3, 8) and for the northern movements (4, 7), respectively. When heavier traffic flows emerge in the southern movements, 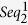 should be selected for the second control ring; On the contrary, 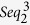 should be selected to alleviate the traffic flows in the northern movements when they become heavier than those in the southern movements. Besides, when traffic flows in both of the opposite directions are approximately equivalent, 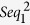 or 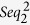 can be adopted, since they simply allocate identical green time for the movements in the opposite directions. In the state space of the dynamical system described by the attractor selection model, there exist two different attractors where one of the metabolic phenotype variables usually overweighs the other. In a non-adaptive attractor state, the two metabolic phenotype variables are approximately equivalent. Accordingly, to match alternative phase sequence solutions with various attractors, we define the following specific rules:

1. *For the first ring*: when 
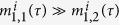
, 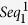 is selected; otherwise, when 
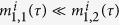
, 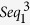 is selected. Additionally, when the state variables are approximately equal to each other, or, in other words, fluctuating around the same level, 
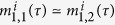
, we will choose 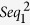 for the first ring.
2. *For the second ring*: when 
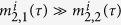
, we choose 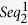 for the second ring, and 
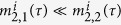
 leads to 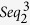. When 
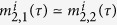
, we will select 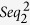.

For practical implementation, we discretise the attractor selection model and exploit the discretised formulation to dynamically update the metabolic phenotype variables, 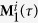 and 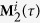:


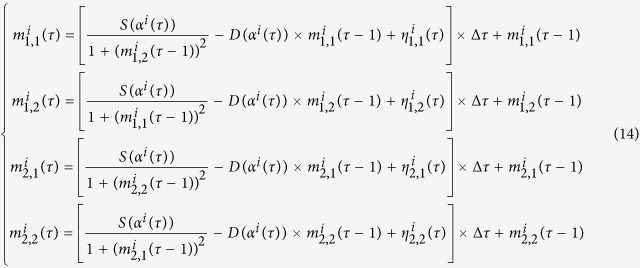


Δ*τ* is the size of the time step between the successive instants *τ* − 1 and *τ*, and it should be sufficiently small in order to ensure the accuracy of the approximation of a continuous model with a discrete-time version. We note that the time instant *τ* is essentially different from the aforementioned *t*; the former is used to indicate the moment when the signal controller operates the phase switching and updates the state of the intersection in real time, while the latter indexes the iterator at which the discrete differential equations (14) are calculated. The smaller Δ*τ* is, the higher the time resolution of the discrete model (14). Indeed, Δ*τ* should be much smaller than Δ*δ*, i.e. Δ*τ* ≪ Δ*δ*. 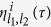 (*l*_1_, *l*_2_ = 1, 2) is defined as an independent white Gaussian noise at the time instant *τ*. Similar to the attractor selection model (9), *α*^*i*^(*τ*) is conceptualised as the cellular activity. The cellular activity parameter, *α*^*i*^(*τ*), is an indicator of fitness of the current signal control solution in the dynamically changing environment. We can model it as a function of the metabolic phenotype variables, 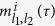, and the available nutrients 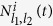. Specifically, inspired by the cellular growth rate model given in (10), we design a discretised form to update *α*^*i*^(*τ*):


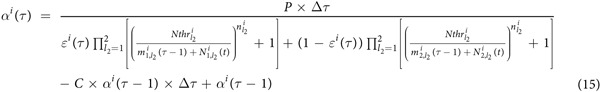


where *ε*^*i*^(*τ*) is a binary indicator, which is set to 1 when the signal controller is in the planning horizon for the first control ring and to 0 when the signal controller is in the planning horizon for the second ring. To be specific, we note that when the signal controller stays at phase 3, during which the movements 2 and 6 are on the green light, the controller should make a decision about the phase switching sequence for the second ring before actual implementation (see Fig. 1(C)). Similarly, when it comes to the phase 7, the controller has to plan the phase sequence for the first ring ahead of time (see Fig. 1(C)).

Based on equations (14) and (15), we can realise an online computation, in which the metabolic phenotypes 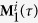 and 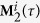, and the cellular activity *α*^*i*^(*τ*), are updated in real-time similar to the adaptive behaviour of a cellular gene network (see the mathematical model system in Fig. 1(F)). When the time instant *τ* comes to the end of a planning horizon, i.e. the end of the phases 3 or 7, we map the attractor of the dynamic system to a solution for the phase sequence selection according to the aforementioned rules 1 or 2. The discrete-time updating equations (14) and (15) can lead to an autonomous and adaptive signal control algorithm for each intersection of the traffic network. The corresponding simplified control logic and the time horizons for operating phase switching and for running the algorithm are illustrated in Fig. 1(G,H), respectively. In the bio-inspired signal control algorithm, the phase-switching for each intersection, is performed at successive time instants: (…, *t* − 1, *t*, *t* + 1, …), and this algorithm is carried out by implementing a synchronous update process in our simulations. Nevertheless, it is worth mentioning that because any intersection with the algorithm does not require the control information of other intersections but only needs the information from the traffic environment, the intersection can operate the algorithm in a fully distributed and asynchronous manner in real-life scenarios. We set the duration of each phase as the time interval Δ*δ*, such that the possible maximum length of a signal cycle is (3 + 3) × Δ*δ* and the possible minimum length is (2 + 2) × Δ*δ*. In a signal cycle, when the controller runs the phase 3 (7), it starts to plan the phase-switching sequence for the second (first) ring, namely, calculating equations (14) and (15). Hence, the phases 3 and 7 are considered as two different planning horizons in a signal cycle. In each planning horizon, the number of updating iterations is *IterNum* = *ceil*(Δ*δ*/Δ*τ*) (*ceil*(·), a function that rounds the value of an input to the nearest integer not less than the input). This indicates that the calculation of equations (14) and (15) is simply performed during phases 3 and 7 in a series of signal cycles, and when the controller stays at other phases, the state variables and the cellular activity are fixed. Once selection of an appropriate phase sequence solution is achieved according to the aforementioned Rules 1 or 2 at the end of a planning horizon (phase 3 or 7), the selected phase switching sequence is implemented for the next control ring.

## Additional Information

**How to cite this article**: Tian, D. *et al.* From Cellular Attractor Selection to Adaptive Signal Control for Traffic Networks. *Sci. Rep.* **6**, 23048; doi: 10.1038/srep23048 (2016).
